# Sclerosing angiomatoid nodular transformation of the spleen mimicking metastasis of melanoma: a case report and review of the literature

**DOI:** 10.1186/s13256-017-1400-6

**Published:** 2017-09-03

**Authors:** Ilhan Demirci, Horst Kinkel, Dirk Antoine, Marc Szynaka, Bernd Klosterhalfen, Susanne Herold, Hermann Janßen

**Affiliations:** 1 Department of General, Visceral, Vascular and Thoracic Surgery, Hospital of Düren, Düren, Germany; 2 Department of Gastroenterology, Hepatology and Diabetology, Hospital of Düren, Düren, Germany; 3 Department of General, Visceral and Thoracic Surgery, Hospital of Leverkusen, Leverkusen, Germany; 4 Department of Radiology, Hospital of Düren, Düren, Germany; 5 Institute of Pathology, Hospital of Düren, Düren, Germany; 6 Department of Internal Medicine II, University of Gießen Lung Center, Gießen, Germany

**Keywords:** Sclerosing angiomatoid nodular transformation of the spleen, Contrast-enhanced ultrasound, Mimicking, Metastasis of melanoma, Splenectomy

## Abstract

**Background:**

Sclerosing angiomatoid nodular transformation is a benign disorder of splenic tissue and is often mistaken as a potentially malignant entity in the diagnostic process. To the best of our knowledge, this is the first report of sclerosing angiomatoid nodular transformation mimicking metastasis of melanoma in the literature.

**Case presentation:**

A 43-year-old white man presented with a newly found splenic mass 4 years ago to our Department of Gastroenterology. He was diagnosed as having a superficial spreading malignant melanoma localized at his left instep 7 years ago and was successfully treated with radical local resection. Several diagnostic procedures were conducted. Ultrasound showed a hypoechoic lesion in the inferior pole of his spleen with a diameter of 2 cm, blurred boundaries, and inhomogeneous interior pattern. Contrast-enhanced ultrasound was inconclusive and showed only discrete contrast enhancement of the lesion with accentuated nodule-like enrichment of the boundaries in the arterial phase. Computed tomography and magnetic resonance imaging scans showed two splenic lesions which were highly suspicious of metastasis. Magnetic resonance imaging of his head was inconspicuous. Bone scintigraphy showed no abnormal results. Fine-needle aspiration indicated metastasis of the above-mentioned malignant melanoma. We conducted a laparoscopic splenectomy. His intraoperative and postoperative course were uneventful. In contrast to the result of the fine-needle aspiration, the presence of metastasis of melanoma could not be confirmed. Histological analysis revealed nodule-like arrangement of fibroblasts with low cell density and a predominance of dilated capillaries, indicating sclerosing angiomatoid nodular transformation of the spleen.

**Conclusions:**

There are no preoperative diagnostic imaging procedures which can definitely differentiate sclerosing angiomatoid nodular transformation from malignancies in cases of morphological and immunophenotypic variations of the specimen. Morphological and immunophenotypic variations of the specimen represent a diagnostic challenge and can mimic malignoma. As reported in our case, the specimen obtained by ultrasound-guided fine-needle aspiration led to the diagnosis of metastasis of melanoma. Splenectomy is often conducted due to a splenic mass suspicious of malignoma as described in our case or with unknown valency in different diagnostic imaging procedures.

## Background

Sclerosing angiomatoid nodular transformation (SANT) as a particular lesion of the spleen was initially described by Martel and colleagues in 2004 [1]. It is generally assumed that many of these lesions were previously classified as inflammatory pseudotumors, hemangiomas, hamartomas, inflammatory myofibroblastic tumor, or exuberant granulation tissue [2–4]. Thus, an accurate statement regarding the prevalence of this entity is hardly possible. To the best of our knowledge, this is the first report of a SANT which was diagnosed in association with a suspected melanoma metastasis of the spleen. In our opinion, the presented case is instructive. Until now, 135 cases of SANT of the spleen have been reported. The mean age is 42.5 years (range 11 to 82 years) with a female to male ratio of 1.18:1.

## Case presentation

**Fig. 1:**
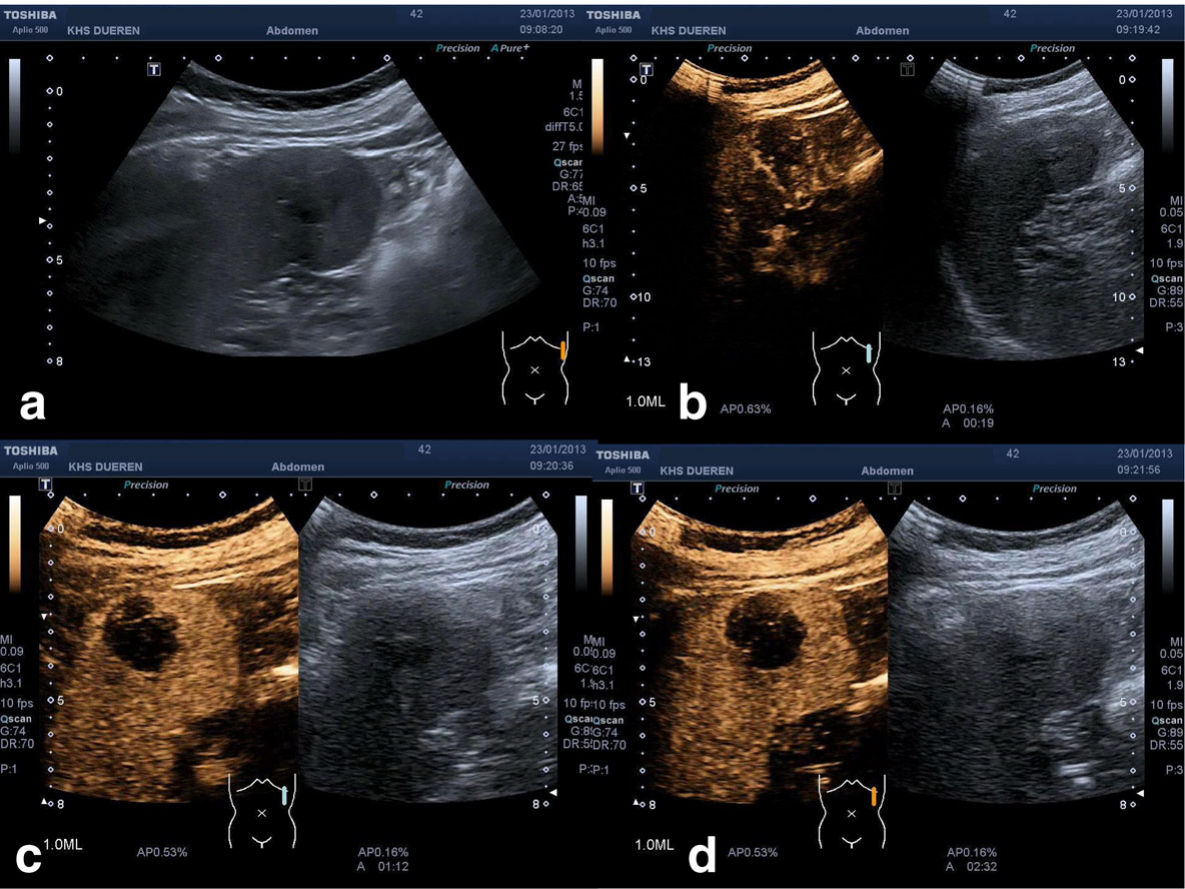
**a** B-mode ultrasound picture of the lesion. **b**, **c**, **d** Contrast-enhanced ultrasound image 19, 72, and 152 seconds after injection of 1 ml SonoVue®

**Fig. 2:**
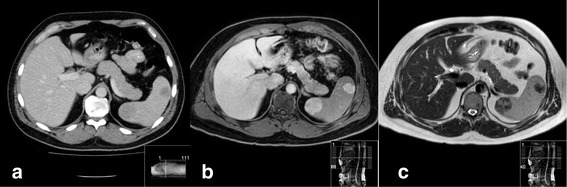
**a** Computed tomography scan of the abdomen showing two hypodense lesions of the spleen. **b**, **c** Magnetic resonance imaging of the abdomen showing the two lesions in T1 and T2 mode, respectively

**Fig. 3:**
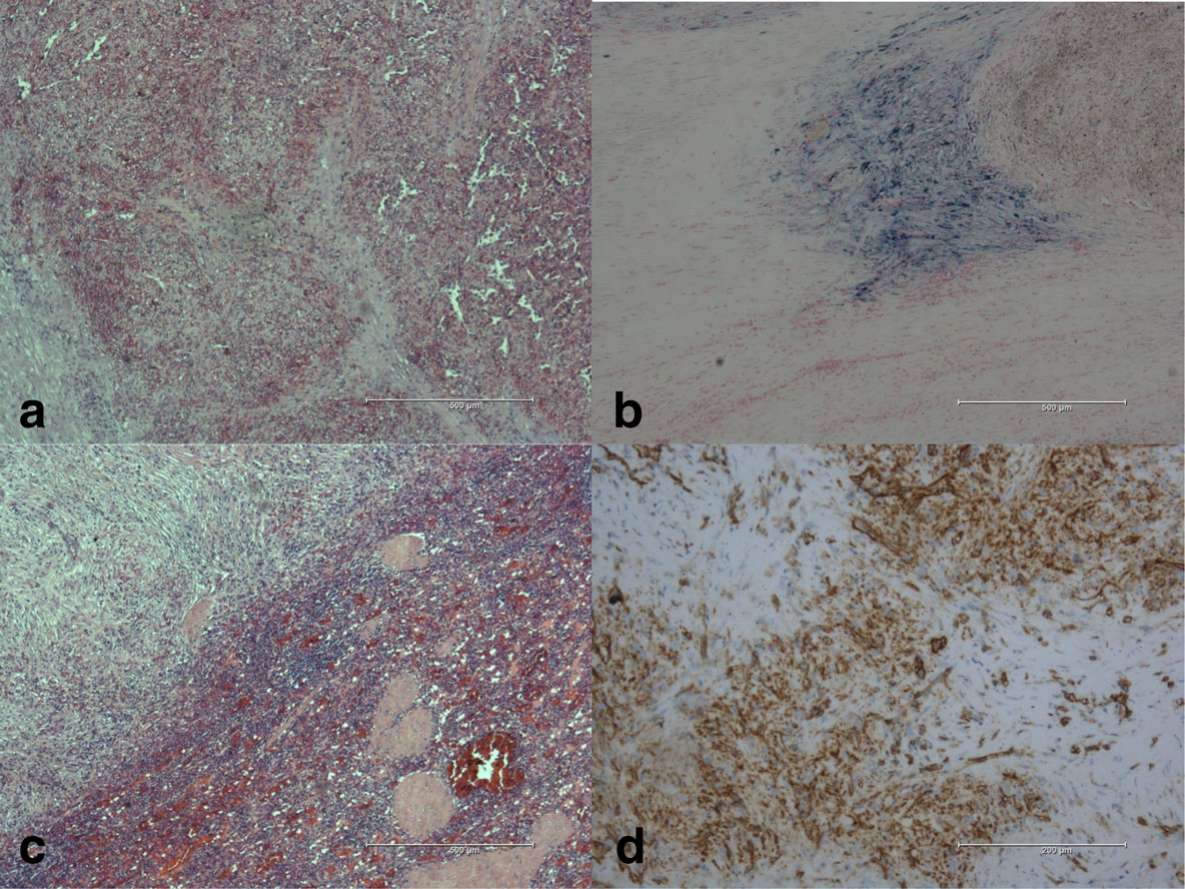
**a** Overview shot with hematoxylin and eosin staining. **b** Iron staining of hemosiderin. **c** Nodule-like arrangement of mesenchymal cells using hematoxylin and eosin staining. **d** Immunohistochemical staining of CD31

A 43-year-old white man presented with a newly found splenic mass 4 years ago to our Department of Gastroenterology, Hepatology and Diabetology. The splenic lesion was found by his general practitioner in a routine ultrasound examination of his abdomen which was conducted 8 weeks before he presented to our Department of Gastroenterology, Hepatology and Diabetology. He was diagnosed as having a superficial spreading malignant melanoma localized at his left instep 7 years ago and was successfully treated with radical local resection (pT1a, Clark-level II, 0.2 mm thickness, R0). He was concerned about a possible relapse of the above-mentioned malignant disease and agreed to further diagnostic procedures and treatment. Anamnesis of current ailments and physical examination were inconspicuous. His family and psychosocial history had no abnormalities. There were no abnormal laboratory values. In the following 3 weeks, diagnostic procedures such as esophagogastroduodenoscopy, ultrasound, contrast-enhanced ultrasound (CEUS; SonoVue®), computed tomography (CT), magnetic resonance imaging (MRI), bone scintigraphy, and ultrasound-guided fine-needle aspiration (FNA) were performed to rule out metastasis. The esophagogastroduodenoscopy revealed a discrete chronic type-C-gastritis, which was treated accordingly with proton pump inhibitors. Ultrasound showed a hypoechoic lesion in the inferior pole of his spleen with a diameter of 2 cm, blurred boundaries, and inhomogeneous interior pattern. CEUS (SonoVue®) was inconclusive and showed only discrete contrast enhancement of the lesion with accentuated nodule-like enrichment of the boundaries in the arterial phase (see Fig. 1). A contrast-enhanced CT scan showed two hypodense lesions of 2.3 cm and 3.5 cm in diameter, which were highly suspicious of metastasis. Contrast-enhanced MRI of his upper abdomen also revealed two suspicious lesions of 2.5 cm and 4.0 cm in diameter, which were hyperintense in T1 and inhomogeneous and hypointense in T2 (see Fig. 2). MRI of his head was inconspicuous. Bone scintigraphy showed no abnormal results. The first FNA was inconspicuous. The second one contained atypical, hyperchromatic, and polymorphic cells and therefore was highly suspicious of malignoma. Immunohistochemical analysis of the specimen revealed overexpression of HMB45, S100, and vimentin, strongly indicating metastasis of the above-mentioned malignoma. The case was presented and discussed at our local tumor board. A splenectomy was recommended. Two weeks prior to surgery, he was vaccinated against *Streptococcus pneumoniae*, *Haemophilus influenzae*, and *Neisseria meningitidis* according to national recommendations of the Robert Koch Institute. We conducted a laparoscopic splenectomy approximately 6 weeks after his initial presentation. His intraoperative and postoperative course were uneventful. He recovered well from surgery and was discharged from our hospital 5 days after the procedure. The obtained spleen was examined by our Institute of Pathology. In contrast to the result of the second FNA, the presence of metastasis of melanoma could not be confirmed. The spleen contained two suspicious lesions of 3.5 cm and 1.7 cm in diameter. Histological analysis revealed a nodule-like arrangement of fibroblasts with low cell density and a predominance of dilated capillaries, indicating SANT of the spleen (see Fig. 3). The histological specimen was sent to a reference pathologist (Prof. Rosenwald, University of Würzburg, Germany) for further immunohistochemical staining and validation of our diagnosis. The following immunohistochemical analysis showed positive expression of CD31 and CD34 in capillaries with no expression of CD8, CD20, and Ki67. The diagnosis of SANT of the spleen was verified. Follow-up care is carried out by our Department of Gastroenterology, Hepatology and Diabetology. Until now, no further abnormalities could be detected. For an overview of the medical history timeline, see Table 1.

**Table 1:** Medical history timeline

Dates	Relevant medical history
02/2010	Superficial spreading malignant melanoma localized at left instep, successfully treated with radical local resection (pT1a, Clark-level II, 0.2 mm thickness, R0)
01/2013	First description of a suspicious splenic lesion found in a routine ultrasound examination of the abdomen conducted by his general practitioner.
03–04/2013	Admission of the patient to our hospital. Esophagogastroduodenoscopy, ultrasound, CEUS (SonoVue®), CT, MRI, bone scintigraphy, and ultrasound-guided FNA were carried out. The immunohistochemical results of the FNA led to the initial diagnosis of metastases of malignant melanoma.
04/2013	Case presentation and discussion at our local tumor board. Splenectomy was recommended. The patient was vaccinated against *Streptococcus pneumoniae*, *Haemophilus influenzae*, and *Neisseria meningitidis* 2 weeks prior to surgery.
05/2013	Laparoscopic splenectomy, uneventful postoperative course. Discharge of the patient after 5 days.

## Discussion

The current etiology of SANT remains unknown. Martel *et al*. suggested a non-neoplastic stromal proliferative process in splenic red pulp tissue with accompanied nodular and later on vascular transformation [1]. Li and colleagues assumed that positive staining for CD38 could indicate active phagocytic process in association with increased splenic activity [5]. Weinreb and colleagues described a case of SANT with concurrent Epstein–Barr virus (EBV) infection and stated the assumption that a subset of SANT may be related to inflammatory pseudotumor of the spleen [6].

SANT lesions are characterized by single or multiple sclerosing angiomatoid nodules with cirrhosis-like architecture without structured red and white pulpa, prominent intranodal fibrotic septa, and an accompanying inflammatory infiltrate. There is a clearly visible sclerotic zone adjacent to the normal splenic tissue, no atypical cells, and only minimal proliferation activity. All three vessel types of the normal spleen can be detected in SANT. However, one can observe a significant predominance of capillaries (CD8−, CD31+, CD34+) in comparison to sinusoids (CD8+, CD31+, CD34−) and small veins (CD8−, CD31+, CD34−) [7]. Recently published studies noted a predominance of IgG4-positive plasma cells in SANT [8, 9]. Upregulated diffuse expression of CD30 and a focal CD68-positive staining pattern seem to be quite common and can be helpful in the diagnostic process [10, 11]. Overexpression of HMB45, vimentin, and S100 is known to be strongly associated with malignant melanoma, although variations in the immunohistochemical expression pattern are common [12, 13]. Vimentin and S100 seem to be highly specific for malignant melanoma, whereas HMB45 is not and is also overexpressed in malignant angiomyolipoma of the liver [14] and pulmonary lymphangiomyomatosis [15]. It is also important to note that positive stainings for CD31, CD34, and vimentin are also found in epithelioid angiosarcoma of bone and soft tissue [16]. Morphological and immunophenotypic variations represent a diagnostic challenge. This extends the list of possible differential diagnoses which must be taken into account and makes further investigations and procedures like splenectomy necessary, as reported in our case.

Most patients were described as asymptomatic in the literature. Only a minority seemed to exhibit abdominal pain, splenomegaly, leukocytosis, polyclonal gammopathy, or other concurrent conditions such as: chronic lymphocytic leukemia; choroidal melanoma; carcinomas of lung, gastrointestinal tract, or kidneys; or deviating blood values [1, 2, 17]. Therefore it is not surprising that the majority of described cases can be classified as incidentaloma.

CT and MRI normally show hypodense/hypointense, solitary splenic lesions, but do not aid in differentiating malignant and benign entities. Conventional abdominal ultrasound most frequently shows a solitary hypoechoic lesion. In clinical practice, it is hardly possible to distinguish highly vascularized masses of the spleen according to their malignant potential or origin [18]. Karaosmanoglu and colleagues recently described the so-called spoke-wheel pattern in CT and MRI as an important imaging clue for making the correct diagnosis of this benign lesion [19]. However, the diagnostic value of this pattern remains unclear, since it does not seem to be present on a regular basis [20]. Diffusion-weighted imaging (DWI) in MRI could be a promising and useful tool in distinguishing SANT from other pathologic entities. However, there are only a few studies regarding this imaging modality [21].

Conventional abdominal and color Doppler imagings do not provide any further differential diagnostic information. SANT lesions may appear hypoechogenic or hyperechogenic and display a lack of consistency in boundaries and shapes [22]. CEUS is known to be a useful tool in delineation of splenic lesions. It can help in differentiating benign and malign tumors [23]. Gutzeit and colleagues reported a predominantly vascularization-conditioned hypoechoic halo artefact and a spoke-wheel pattern on CEUS imaging in a case of SANT [24]. Although CEUS is a useful method and can provide a lot of additional information regarding vascularization and morphology, not every case of SANT seems to show these features in CEUS [22]. Watanabe and colleagues stated in a recently published work that Sonazoid® can dynamically visualize blood flow distribution in the tumor in the vascular phase and detect tumors in the postvascular phase, because it is taken up by reticuloendothelial cells of the spleen [25]. This feature distinguishes Sonazoid® from SonoVue®. Thus, Sonazoid® contrast agent may be useful for exclusion of malignant tumors such as lymphoma and metastatic tumors [25]. Positron emission tomography (PET)/CT scans seem to be of no further use in distinguishing SANT from other malignancies, since there are also reports of PET-positive cases [26, 27]. PET/CT is known to have a high accuracy in detection of melanoma metastasis [28, 29]. There are reports of PET-negative cases of melanoma metastasis in the literature which are noteworthy. Thus and in regard of the before-mentioned highly suspicious FNA specimen, our local tumor board did not advise conducting a PET/CT, since it would have had no significant clinical impact on management decisions [30–32]. Image-guided FNA can be useful in the process of diagnostic evaluation and averting splenectomy but carries the risk of bleeding, non-diagnostic biopsies, and needle tract seeding in cases of malignoma. In our case, the result of the FNA was suspicious for metastasis of melanoma and led us to conducting a splenectomy. Either laparoscopic or conventional splenectomy are to date the most conducted techniques of obtaining a specimen for definitive histopathological studies and, furthermore, in cases of malignancy, for treatment. Correct preoperative diagnosis of this benign abnormality can be difficult, although DWI, CEUS, and FNA seem to be promising and useful tools.

Splenectomy, either as a laparoscopic or a conventional procedure, is often conducted due to the observation of a splenic mass with unknown valency in different diagnostic imaging procedures. Furthermore, most surgeons will tend to perform splenectomy if an observed splenic mass shows a tendency of growth in follow-up controls [21]. In this regard, splenectomy can be referred to as “curative”. It should always be kept in mind that possible life-threatening complications such as overwhelming post-splenectomy infection (OPSI) may occur. Therefore, perioperative vaccination should be mandatory.

## Conclusions

SANT is a benign disorder of splenic tissue and is often mistaken as a potentially malignant entity in the diagnostic process. There are no preoperative diagnostic imaging procedures which can definitely differentiate SANT from malignancies in cases of morphological and immunophenotypic variations of the specimen, although DWI, CEUS, and FNA seem to be promising and useful tools. However, there are only a few studies regarding DWI findings. Morphological and immunophenotypic variations of the specimen represent a diagnostic challenge and can mimic malignoma. This extends the list of possible differential diagnoses which must be taken into account and leads to further investigations and procedures like splenectomy, as reported in our case. As reported in our case, the specimen obtained by ultrasound-guided FNA led to the diagnosis of metastasis of melanoma. To the best of our knowledge, this is the first report of SANT mimicking metastasis of melanoma in the literature.

## Abbreviations

CEUS: Contrast-enhanced ultrasound
CT: Computed tomography
DWI: Diffusion-weighted imaging
EBV: Epstein–Barr virus
FNA: Fine-needle aspiration
MRI: Magnetic resonance imaging
OPSI: Overwhelming post-splenectomy infection
PET: Positron emission tomography
SANT: Sclerosing angiomatoid nodular transformation
